# A reverse genetic screen for genes involved in ciliary formation and function

**DOI:** 10.1186/2046-2530-1-S1-P35

**Published:** 2012-11-16

**Authors:** D Moore, P zur Lage, G Gallone, FE Rodger, AP Jarman

**Affiliations:** 1 University of Edinburgh, UK

## 

*Drosophila* provide an excellent opportunity to study cilia formation and function as the sensory neurons are the only ciliated somatic cells, and so cilium mutations are recognisable by sensory behavioural defects. A reverse genetic screen was conducted to implicate candidate genes in ciliary processes. Candidates were selected from a chordotonal sensory neuron transcriptome analysis (Cachero et al., 2011) and refined by consideration of gene ontology, existence of human homologues, and participation in protein-protein interaction networks (Gallone et al., 2011). P element insertions and Gal4/UAS induced RNAi were used to disrupt gene function. Candidate genes were screened using a climbing assay to select for proprioceptive and gravitactic deficiency, indicating disrupted chordotonal neuron function. Positive hits from the screen are being investigated by observing the ciliary dendrite structure of pupal antennae and larval chordotonal neurons. Positive candidates are also tested for fertility defects due to disrupted flagellum formation during spermiogenesis. Our analysis has identified several novel conserved ciliary genes. Particular attention has been given to genes regulated by the transcription factor fd3F, which is required for the transcription of several genes involved in cilium motility.

